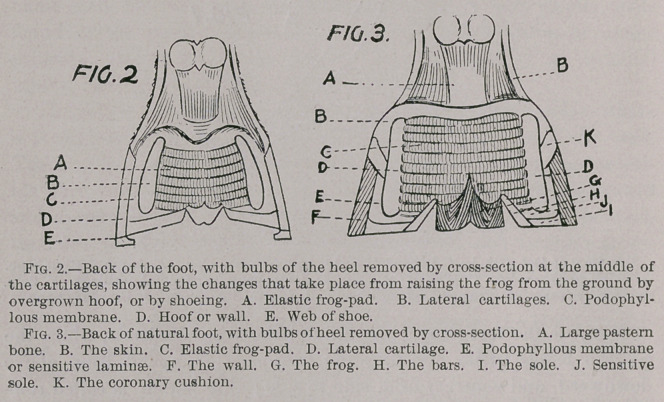# The Foot of the Horse1Read before the meeting of the Wolverine Veterinary Medical Association, December, 1902.

**Published:** 1903-02

**Authors:** L. L. Conkey

**Affiliations:** Grand Rapids, Mich.


### THE FOOT OF THE HORSE.[1]

By L. L. Conkey, D.V.S.,
GRAND RAPIDS, MICH.

The subject which I have chosen for presentation to you at this meeting is by no means a new one, yet we feel that there are many people to-day, raising, breaking, and driving horses that know but little of the true functions of the *horse's foot*, other than it is an organ of locomotion.

Without a doubt, more horses are retired from work annually on account of their feet going wrong than from all other causes combined. This is due to three primary causes:

1. From the lack of a proper knowledge and understanding of the anatomical and physiological functions of the foot on the part of the breeder.
2. Early and indiscriminate shoeing.
3. The perversion of nature, as well as a lack of ability and understanding on the part of the horseshoer who does the work.

To correct those errors, we must begin with the first great cause, which we say is the lack of a knowledge and understanding of the anatomical and physiological functions of the foot, the remedy for which is *education*, and to educate the public along these special lines we must place the subject before them in plain, comprehensive language, avoiding technical terms as much as is possible; therefore we have endeavored to use the most common language at our command in preparing this paper.

The foot of the colt at birth is largest at the coronary band, junction of the hoof and hair, tapering downward in sort of a cone, terminating in a soft cartilage, that it may not injure the maternal organ, before or during birth. This cartilaginous portion soon wears off, leaving the hoof at first cylindrical, which now gradually changes in form for some weeks, when it assumes the shape so characteristic of the type. The lower border of the wall of the hoof is naturally larger than is the upper; at the lower part of the heels

[1] Read before the meeting of the Wolverine Veterinary Medical Association, December, 1902.

the wall is turned in forming the bars, each bar joining its fellow near the centre of the sole at the point of the frog, leaving a triangular space in the back part of the sole in which is lodged the non-sensitive frog. The sole is situated in the lower border of the wall, in an arched position, so that on hard, level surfaces it takes no bearing.

The wall has a glossy covering, called the periople; this covering maintains the whole wall at a uniform degree of softness and toughness, so essential to the elasticity and life of the hoof.

On each projection of the sole which is embraced between the bars and wall of the hoof, is the well-known seat of corns; the name *corn*, however, is a misnomer, as the dark, ecchymotic spots found at the point mentioned is the result of a bruise or undue pressure, and is really the external manifestation of an internal injury, having no similarity to the human corn.

The sole is of uniform thickness, except at its outer border, where it is slightly thickened, forming a firmer attachment with the wall. The frog is a triangular mass of soft and comparatively indestructible fibre, with its apex pointing forward, the fibres doubling inward and upward, form a fissure below and a wedge pointing up between the bulbs of the heels above; thus, we have the non-sensitive structure of the horse's foot. The sensitive structure of the foot embraces the pedal, navicular, and small pastern bones; the extensor and flexor tendons; the inferior or lower sesamoid ligaments, the lateral cartilages, the sensitive sole, sensitive frog, elastic frog-pad or cushion, arteries, veins, and nerves.

By observing the accompanying pen sketches, which are the handiwork of the author, it will be seen that the pedal bone occupies the front part of the foot; and it is covered with a series of soft structure, terminating in the podophyllous membrane, a leaf-like arrangement, dove-tailed into a like arrangement on the inner side of the wall of the hoof. There are about 500 of these leaves, extending from the coronary band down the length of the hoof; they are non-elastic or unyielding.

The os pedis, or pedal bone, is situated in the front part of the hoof, extending about two-thirds of the way back (as shown in drawing, Fig. 1); the remainder of the foot is composed of soft structures; the lateral cartilages, which form the base or bulbs of the heels, are covered on their lower outer surface with podophyllous membrane, giving a firm attachment to the wall. These lateral cartilages are bound together by strong ligaments, to prevent undue spreading of the heels as the great weight of the animal falls suddenly upon them, as in jumping or running.

These ligaments form a network between the cartilages, the meshes of which are filled with elastic material; combining, they form the elastic *frog-pad* (see Fig. 3), and also give form and shape to the bulbs of the heels. Thus the elastic frog-pad is found between the cartilages behind the pedal bone, above the frog and below the pastern bones, so that pressure, when the frog rests upon the ground, compresses the frog-pad, causing it to expand latterly—that is, from side to side—to the full extent of the ligaments; this movement is called *expansion.*

Side view of foot with half of the wall and membrane removed. A. Large pastern bone. B. Small pastern bone. C. Wall of the hoof. D. Pedal bone. E. Lateral cartilage. F. Sole. G. Sensitive sole. H. Frog-pad between heels. I. Lateral ligaments. J. Coronary band.

Now, as the foot leaves the ground the frog descends, while the pastern bones raise, allowing the frog-pad to expand from above downward, and contract from side to side; this action is called *contraction.* Therefore, it will be seen that in the natural foot the frog taking a ground bearing, we have expansion and contraction of the hoof at each and every step the animal takes.

Now, let us go a little deeper into the physiological functions of the horse's foot. We have stated, and have a hoof, too, for inspection, that the sole of the horse's foot is *arched*, or concaved on its ground surface, so that on level surfaces it takes no bearing; we also say that we have expansion of the heels at each and every step. The sole being round and concave on its under side, it will surely be more or less *cone*-shaped on its upper side; when you increase the circumference of a cone, just in that proportion do you diminish its concavity. Is this not true? In other words, the expansion and contraction at the base of the horse's hoof produces the same (although limited) action as that produced upon the diaphragm by the expansion and contraction of the muscles of the chest; hence the first action of the sole is to recede from the foot in a degree proportionate to the expansion at the heel, forming a vacuum which is immediately filled with blood; as the heel always—in a healthy condition—touches the ground first at any pace, as has been demonstrated by instantaneous photography, so always does this action take place; as the animal body is carried forward, the foot becomes weighted, the blood is forced into the upper veins from whence it came; the frog recedes and contraction follows.

This is nature's pneumatic action, not witnessed by us in any other animal, and we claim that this action is essential to the life and well-being of the horse's foot, and can be maintained only through natural conditions—*frog pressure.* (See Fig. 3.)

If you please, gentlemen, we will now go a little farther, and inquire into the changes brought about by allowing the hoof at the heels to become overgrown, raising the frog from off the ground. We have said that anatomy shows the lateral cartilages to be tied together with strong ligaments, the office of which is to prevent undue spreading of the hoof at the heels, as the frog is forced up between them. Now, then, we say the hoof at the heels has been allowed to become so overgrown that the frog no longer has a bearing. What is the result? Simply this, the ligaments that bind the cartilages together are converted into a sling or swing, in which the pastern bones are hung, and when the pastern bones are weighted the frog descends; the frog-pad is now a cushion in a swing, which also descends, drawing the cartilages together at every step taken. (See Fig. 2.)

Completely reversing the laws of nature, and what is the result? A stilty gait. Can you see any difference between the action of this overgrown hoof which raises the frog from off the ground and the shoe with long calkings? There is absolutely no difference. No man on earth can shoe a horse using a calked shoe, except that he injures the foot more or less.

This reversal of the natural functions of the foot accounts for the condition found in the hoofs of nearly all old horses, viz.: long, deep, narrow heels, a hard, dry, wasted frog and contracted feet.

The foot of the suckling colt, but a few weeks old, requires careful attention; those having their care, should at frequent intervals pick up the feet, examine the bars, frog and general condition of the foot. Should one side be found higher than the other, dress it down; if both heels are long and the frog high off the ground, dress them down to allow the frog a ground bearing. Should the toe become overgrown, it, too, must be reduced. This is the age of the animal when we would recommend *shaping the foot to the eye.* If the heels are allowed to become overgrown, the frog thrown into disuse, it will surely dry up and waste away; contraction, thrush and, goodness only knows what else will follow. Again, if *one heel* is allowed to become overgrown, the opposite side will become distorted, turning outward or bending inward, and a spavin or ringbone is the sequel most liable to follow; while an overgrown toe throws the weight back upon the pastern joints; this, if allowed to remain for any length of time, sets up an irritation, lameness and perchance a ringbone follows. In fact, we are of the opinion that nine-tenths of all ringbones and spavins found on young horses are the results of one or the other of the foregoing conditions.

In the natural foot, it will be observed, that the front two-thirds of the foot is composed of the thickest and strongest part of the hoof, which is reinforced by the pedal bone, rendering it firm and unyielding, while the remaining back portion of the foot is made up of soft, elastic material, designed to ward off concussion as the foot comes in contact with the ground. The colt's foot grows more rapidly than do the feet of horses of mature years; for this reason, the shod foot of the young horse is soon out of balance, and when out of balance, at once starts on the down grade to ruin.

From an anatomical, physiological or functional point of view, it appears to us worse than folly to nail shoes on the feet of any colt before it is four or five years old. Should it be deemed necessary to break and drive a colt, drive it barefoot; should the hoof wear off sufficient to produce lameness, turn it in the pasture field, and nature will do the rest. The feet of all young horses will, unshod, withstand as much hardship as the undeveloped bones and muscles should be called upon to endure.

## Figures and Tables

**FIG.1 f1:**
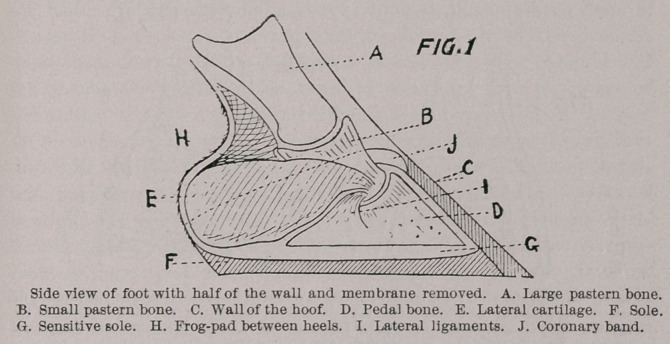


**FIG.2 FIG.3. f2:**